# Carl Jung: a life on the edge of reality with hypnagogia, hyperphantasia, and hallucinations

**DOI:** 10.3389/fpsyg.2024.1358329

**Published:** 2024-03-07

**Authors:** Fatih Incekara, Jan Dirk Blom

**Affiliations:** ^1^Parnassia Psychiatric Institute, The Hague, Netherlands; ^2^Faculty of Social and Behavioural Sciences, Leiden University, Leiden, Netherlands; ^3^Department of Psychiatry, University Medical Centre Groningen, Groningen, Netherlands

**Keywords:** biography, imagery, psychotic disorder, psychoanalysis, schizophrenia spectrum disorder

## Abstract

Whether the Swiss psychiatrist Carl Jung (1875–1961) became psychotic after his mid-thirties is much debated. His recently published *Black Books,* a seven-volume journal, reveal new insights into this debate. Based on a phenomenological analysis of his self-reports in these books and in other writings, we here identify several types of anomalous perceptual experiences: hypnagogic-hypnopompic experiences, hyperphantasia, hallucinations, personifications, and sensed presence. We argue that these experiences were not indicative of a psychotic disorder, but rather stemmed from extremely vivid mental imagery, or hyperphantasia, a condition Jung’s contemporaries and later biographers were unable to take into account because it had not yet been conceptualised. Recently, the degree of vividness of mental imagery and its potential to become indistinguishable from regular sense perception has been the subject of extensive studies. Unknowingly, Jung may have foreshadowed this line of research with his psychoanalytic concept of reality equivalence, i.e., the substitution of an external world for an inner mental reality that he encountered in individuals diagnosed with schizophrenia. There is a need for future research to investigate the possible role of hyperphantasia in psychotic experiences, but to Jung, psychosis was ‘a failure to contain and comprehend’ the content of one’s experiences in the context of one’s own life, whereas he himself did manage to put the content of his perceptual experiences into context, to find meaning in them, and to share them with others - to great acknowledgement and acclaim.

## Introduction

*“For me reality means scientific comprehension. It is, of course, ironical that I, a psychiatrist, should at almost every step of my experiment have run into the same psychic material which is the stuff of psychosis and is found in the insane. This is the fund of unconscious images which fatally confuse the mental patient. But it is also the matrix of a mythopoeic imagination which has vanished from our rational age”* (Jung and Jaffé, 1965, p. 226). Carl Jung, 1916.

At the age of 38, the Swiss psychiatrist Carl Gustav Jung (1875–1961) feared that he had become psychotic. For ten months he had been experiencing catastrophic dreams, hallucinations, and what he himself described as ‘visions’. He struggled to make sense of these sensations, but it was not until August of 1914, after the onset of what was to become known as the Great War, that he interpreted the rivers of blood and dead bodies he had seen in his mind’s eye as truthful premonitions (Jung and Shamdasani, 2020a,b). He nonetheless realised that ‘to the superficial observer’ his experiences might still ‘appear as madness’ (Jung and Shamdasani, 2009, p. 226). Yet, the first claim that Jung was mentally ill did not come from ‘superficial observers’, but rather from his contemporaries in psychoanalytic circles. Sigmund Freud repeatedly wrote to his colleagues Sándor Ferenczi and Karl Abraham that Jung was ‘crazy [*meschugge*]’ (Freud et al., 1993, p. 440). Freud also received a letter from Ernest Jones in which the latter stated that Jung was ‘mentally deranged to a serious extent’ and that he ‘made a paranoiac impression’ (Freud and Jones, 1993, p. 199). Similar such claims continued after Jung’s death. Jung biographer Paul Stern described him as ‘hallucinating ghosts and demons’ while in a state of ‘semi-psychosis’, although he also credited him to be a visionary ‘without being manifestly mad’ (Shamdasani, 2005, p. 73). Deirdre Bair, another Jung biographer, describes how in 1961 Richard F.C. Hull, translator of *The Collected Works of C.G. Jung,* was invited by the psychiatrist to read his *Red Book*, after which he concluded that the then unpublished work was ‘the most convincing proof that Jung’s whole system is based on psychotic fantasies’ and that it was ‘the work of a lunatic’ (Bair, 2003, p. 293). To date, there is an ongoing debate about whether Jung became psychotic. With a careful phenomenological reconstruction of his experiences (see Supplementary material) as derived from the recently published seven-volume *Black Books* (Jung and Shamdasani, 2020a,b,c,d,e,f,g), his other posthumously published *Red Book* (Jung and Shamdasani, 2009), and his *Memories, Dreams, Reflections* (Jung and Jaffé, 1965), we sought to provide revealing insights that may help settle the matter. Based on Jung’s self-reports in these works, we can identify several types of anomalous perceptual experiences: hypnagogic-hypnopompic experiences (hallucinations experienced during the intermediate states of wakefulness and sleep), hyperphantasia (extremely vivid mental imagery), unimodal hallucinations in various sensory modalities, compound hallucinations, personifications, and sensed presence (Blom, 2023).

## Hypnagogia

In a 1959 BBC interview Jung stated that from early childhood onwards his ‘relation to reality was not particularly brilliant’ and that he was ‘often at variance with the reality of things’ (Jung, 1959). Especially between October 1913 and August 1914, he struggled to understand what was real. For example, in December 1913 he awoke from a dream hearing a voice commanding him to shoot himself if he found himself unable to understand it (Jung and Shamdasani, 2020b, p. 175). Like all Swiss military veterans, Jung had a gun, which he kept loaded nearby, and he found the dilemma so pressing that he actually considered ending his life. Another night, he saw a little girl standing in the corner of his room (Jung and Shamdasani, 2020b, p. 206). Months later, he heard an old man speaking in the middle of the night (Jung and Shamdasani, 2009, p. 473). Jung was familiar with such nightly experiences as he had heard similar strange voices in his childhood and had once seen a small girl hovering in his room when he suddenly awoke from a dream during adolescence (Jung and Shamdasani, 2020b, p. 225).

Such *hypnagogic-hypnopompic experiences* are relatively common, with an estimated prevalence of up to 85% (Ghibellini and Meier, 2023). They are considered physiological phenomena that lie within the normal range of perception, even though they have also been reported in individuals with a psychotic disorder (Waters et al., 2016). A phenomenological distinction between hypnagogia on the one hand, and hallucinations indicative of psychotic disorders on the other, is therefore relevant. The cardinal difference that needs to be highlighted, is that hypnagogic-hypnopompic experiences take place during sleep–wake transitions, whereas hallucinations proper are by definition experienced during wakefulness. This even holds true for so-called complex nocturnal visual hallucinations, which take place during brief moments of interrupted sleep (Waters et al., 2024). Hypnagogia may therefore sometimes be hard to distinguish from (sleep-onset) dreams, whereas this does not tend to be the case for hallucinations. An additional difference between hypnagogia and hallucinations is that especially hypnagogic hallucinations tend to be (visual or multimodal) immersive experiences whereas hallucinations are usually experienced against a background of regular sense perception or even embedded in it. Exceptions to this rule of thumb are panoramic hallucinations (which replace the entire sensory input picture) and hypnopompic hallucinations (which may also be experienced superimposed upon, or embedded in, the regularly perceived environment; Blom, 2023).

Jung was well aware of having hypnagogic-hypnopompic experiences without him losing his grip on reality. In April 1919, again in the middle of the night, he saw the face of an old woman and heard all sorts of sounds, relating the face to that of a woman with cancer whom he had met before, corresponding to a multisensory hypnopompic experience, rather than a psychotic hallucination (Jung and Shamdasani, 2020f, p. 199). Jung regarded hypnagogic-hypnopompic experiences as sense-dependent, believing that they were based on earlier retinal-field excitations, perhaps even in a distant past (Jung, 1970, p. 60) – an explanation that has since been rejected (Ghibellini and Meier, 2023). He also hypothesised that these phenomena were closely related to dreams and mental imagery, especially in highly imaginative people. With his own extreme vividness of imagination, Jung may well have had himself in mind when proposing this relationship.

## Hyperphantasia

Only recently, the phenomenon of having a lively imagination, or *hyperphantasia*, is conceptualised and systematically being investigated as a new field of research (Pearson, 2019; Zeman et al., 2020; Milton et al., 2021). With hyperphantasia, people experience such extreme vividness of their mental imagery that it approximates regular visual perception. In the general population, hyperphantasia has an estimated prevalence of 3% (Milton et al., 2021). The vividness of mental imagery is assessed with subjective scales such as the *Vividness of Visual Imagery Questionnaire* (VVIQ), but also with objective techniques such as measuring pupil diameter, the latter being correlated to the brightness of visualised images (Marks, 1973; Pearson, 2019; Zeman et al., 2020). Although it is suggested that vivid mental imagery is linked to schizophrenia spectrum disorders, more research is needed to investigate this (or an association with psychiatric disorders in general) (Sack et al., 2005; Pearson, 2019).

To the best of our knowledge, it was Karl Jaspers who first systematically compared extremely vivid mental imagery, or ‘pathological imagery’ as he called it, with regular mental imagery and perceptual anomalies such as hallucinations (Jaspers, 1968). As he wrote,

“They [pathological images] differ from normal images in their greater sensory concreteness, clarity and detail, their appearance independently of, and even against, the subject’s will, and by the accompanying experience of passivity and receptiveness. On the other hand, they differ both from true hallucination and from normal perception in that they do not appear in external space as perceptions do, but in the internal space in which images also are experienced” (Jaspers, 1968, p. 1319).

A recent study compared phenomenological and neurophysiological aspects of mental imagery and hallucinations and concluded that both are ‘stimulus-independent’ types of perception, mediated by activity in shared association and visual cortices (Waters et al., 2021). As already recognised by Jaspers, imagery and hallucinations differ in vividness and their accompanying sense of reality, with imagery being less vivid, and rarely leading to a loss of one’s sense of reality. However, the authors also point out that extreme vividness of mental imagery may be an exception to this, and that ‘frequent practice can sharpen the perceptual qualities and intensity of imagery’ (Waters et al., 2021, p. 3). Jung appears to have done exactly that. He was in the possession of an extreme vividness of mental imagery that was probably congenital, and developed it further in a systematic manner to which he referred as ‘active imagination’. Through this type of imagination, he claimed to be able to deliberately evoke and capture extremely vivid mental imagery, as documented between 1913 and 1932 in his *Black Books* (see Supplementary material) (Jung and Shamdasani, 2020a,b,c,d,e,f,g). A recent study found an association between hyperphantasia and openness, described as ‘an openness to new experiences, broad interests, and an active imagination, and a likelihood of experiencing both positive and negative emotions more keenly than most people’ (Milton et al., 2021, p. 12). The authors also found that people with hyperphantasia have a more detailed autobiographical memory than those who have less vivid mental imagery (Milton et al., 2021). Jung was indeed able to recall specific lively details from his youth, and mentions that already as a child he was capable of making the picture of his deceased grandfather move by focusing on the frame and thus willing his grandfather to come down the stairs (Jung, 1976, p. 171).

A recent experimental study using computational and neuroimaging data showed that mental imagery, when it becomes vivid enough, may cross a ‘reality threshold’ that makes it indistinguishable from regular sense perception (Dijkstra and Fleming, 2023). Studies have also suggested that individuals with higher degrees of vividness of mental imagery are more likely to experience hallucinations (Dijkstra and Fleming, 2023). Although this is in need of further study, it highlights the relevance of vividness in the field of mental-imagery research.

Despite known theoretical and phenomenological characteristics of imagery, Jung’s experiences of hyperphantasia also show, as a relatively rare extremity of mental imagery, that a phenomenological differentiation between anomalous experiences can be challenging. One night in January 1927, Jung had an extremely vivid mental image of a good friend who had recently passed away. He was not dreaming, because he had already been fully awake for a while. Since he was not sure whether he was merely experiencing an hallucination, he considered getting out of bed and following him to the door. He could barely distinguish between the mental image and reality, and only after due consideration did he follow his friend ‘in his imagination’ as he expressed it, rather than in the physical world (Jung and Shamdasani, 2020f, p. 240). Such experiences of extraordinarily vivid mental images would later lay the foundation for his unique self-experimentation, which he called his ‘confrontation with the unconscious’ (Jung and Shamdasani, 2020a,b,c,d,e,f,g).

## Hallucinations

In the ensuing ‘visions’, as Jung later called them in his *Black Books*, throughout his life he had been summoning up characters that he would subsequently see, hear, and have mental conversations with (see Supplementary material; Jung and Shamdasani, 2020a,b,c,d,e,f,g). Some main figures with whom he entertained such inner dialogues were Elijah, the Old Testament prophet, whom he later identified as the wise old Philemon, and a young Salome, whom he established as being his soul (Jung and Shamdasani, 2009). Jung did not only see these figures in the form of vivid mental images but simultaneously heard their voices with his ears (compound hallucinations) or even felt their voices in his mouth (possibly synaesthesia) (Jung and Shamdasani, 2020a, p. 25). It is indeed reported that people with hyperphantasia experience synaesthesia more frequently than those with less vivid mental imagery (Barnett and Newell, 2008; Zeman et al., 2020).

**Figure 1:**
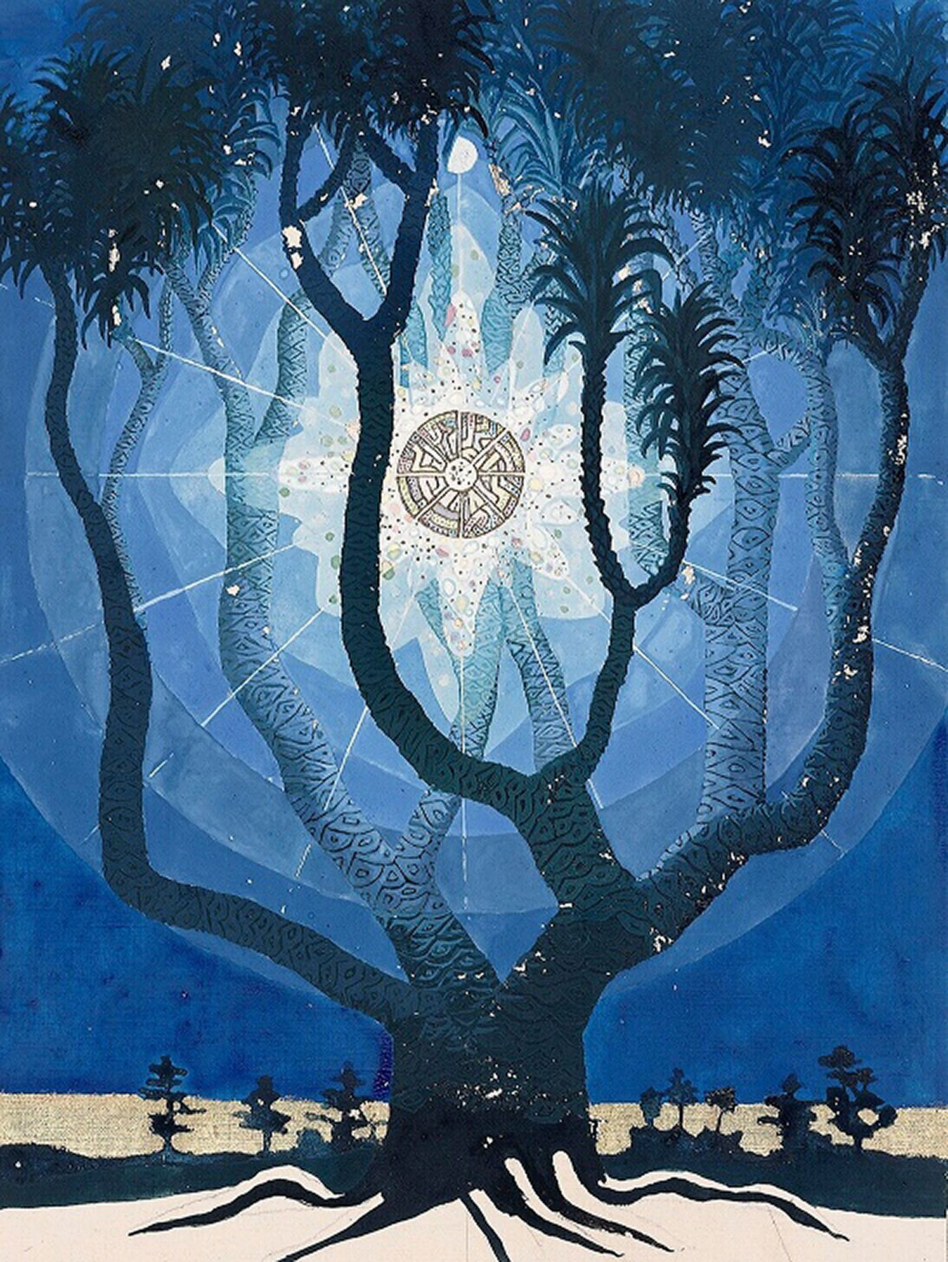
Philosophical tree. ^©^2009 Foundation of the Works of C.G. Jung, Zürich. First published by W.W. Norton & Co, New York, NY. Reproduced with kind permission.

**Figure 2:**
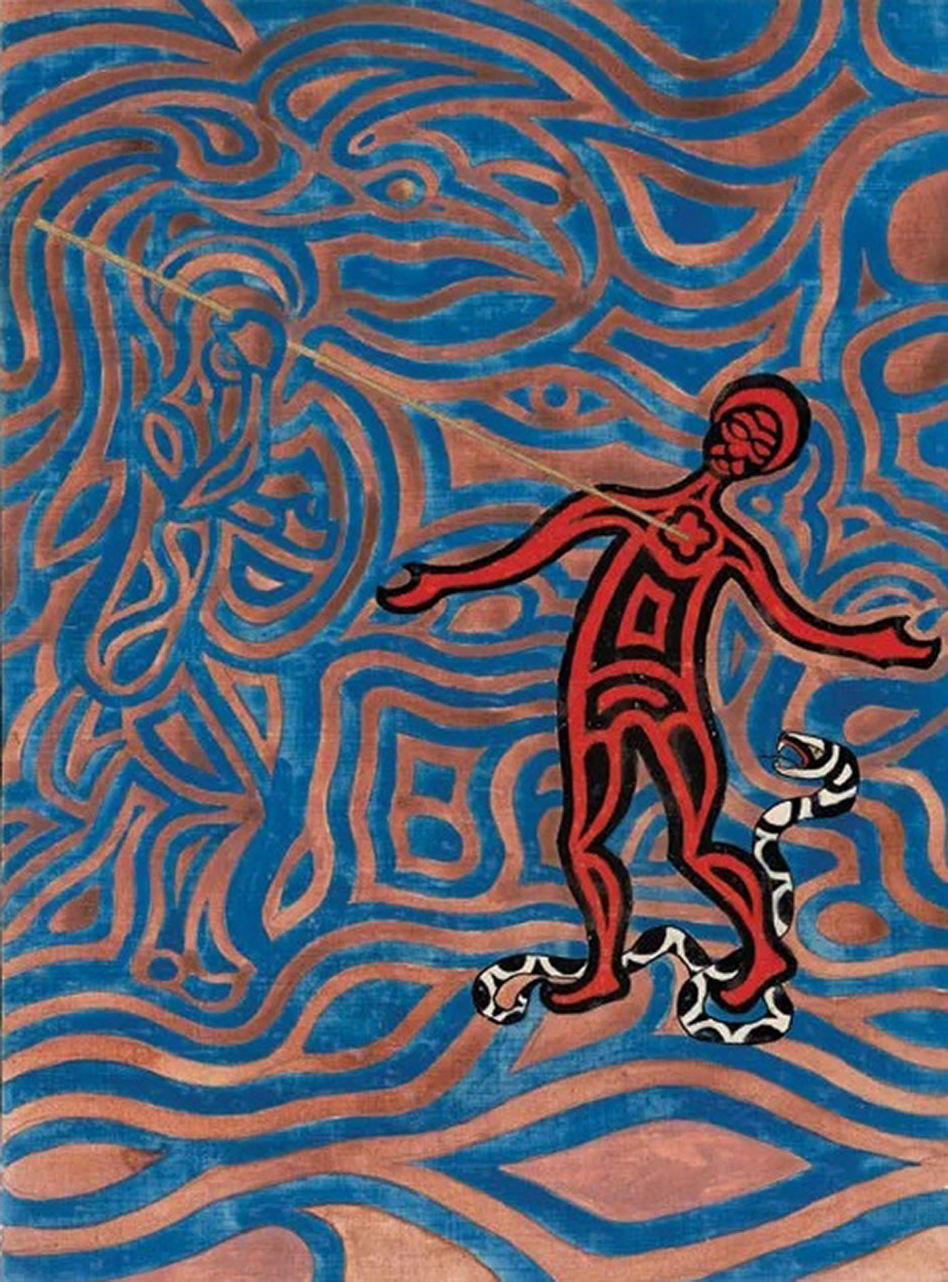
The man of matter. ^©^2009 Foundation of the Works of C.G. Jung, Zürich. First published by W.W. Norton & Co, New York, NY. Reproduced with kind permission.

In 1912, the German chemist Ludwig Staudenmaier introduced the term *personification* to denote such life-like hallucinated projections of human figures (which he himself also experienced) (Blom, 2023). Although Jung and Staudenmaier never seem to have met, they were familiar with each other’s work. Staudenmaier refers to Jung in his book *Die Magie als experimentelle Naturwissenschaft*, while Jung had marked several passages in a copy of Staudenmaier’s book that he possessed (Staudenmaier, 1912; Jung and Shamdasani, 2020a). Staudenmaier, a university professor who in later life was diagnosed with schizophrenia, claimed to have developed a technique to induce auditory, visual, and even compound hallucinations. He recounted how he engaged in dialogues with his own personifications, knowing they were projections, while simultaneously acknowledging them as autonomous beings once he had projected them ‘out there in the world’. So as to access the content of the unconscious, Jung also engaged in self-experimentation, calling his *Black Books* his written unconscious. Besides his written work, Jung produced paintings, sculptures, and wood carvings. The *Red Book*, for instance, not only contains aesthetic calligraphic representations of his visions, but also images depicting his kaleidoscopic mental imagery (Jung and Shamdasani, 2009; Figures 1, 2). However, Jung refused to designate his ‘painted unconscious’ as art, explaining that he merely used artistic means as an instrument to give expression to the nature of his unconscious (Jung and Jaffé, 1965, p. 186).

Jung wandered the edges of reality from time to time, especially when he ‘interacted’ with his projected human characters. These interactions appear to have chiefly occurred at night during hypnagogic-hypnopompic states and on hot summer days in June 1916 in the course of hallucinatory states when he would hear (*auditory hallucinations*), see (*visual hallucinations*), and engage in inner dialogues with his spiritual leader Philemon in his garden (Jung and Shamdasani, 2009, p. 551). Such multimodal hallucinations are traditionally considered rare, although a large study among patients diagnosed with schizophrenia spectrum disorders shows that within that group, they have merely been underreported (Lim et al., 2016). That same year, Jung had an uncanny sensation of a crowd of spirits residing in his house who, according to his own account, had just returned from Jerusalem (*sensed presence*, i.e., the intuitive feeling of some entity nearby that cannot be perceived) (Jung and Jaffé, 1965, p. 225). Later, he could also *see* these spirits and conducted long dialogues with them, eventually concluding that he ‘should return to reality’ as he needed ‘firmer ground for such experiences’ (Jung and Jaffé, 1965, p. 224). In 1944 he briefly lost touch with reality when in a delirium after having fractured a foot and suffering a myocardial infarction. During this episode he temporarily had geometric visual hallucinations, an out-of-body experience, and a near-death experience (Jung and Jaffé, 1965, p. 341).

## Discussion

Based on our phenomenological analysis of the events that Jung reports in his posthumously published works, we identified several anomalous perceptual experiences outside the context of psychopathology. Jung experienced instances of hypnagogia, hyperphantasia, auditory, visual, and tactile hallucinations, compound hallucinations, personifications, and sensed presence, plus an out-of-body and near-death experience while delirious. Retrospective diagnosis is always a knotty issue, but none of these experiences seem to add up convincingly to what we now call a schizophrenia spectrum disorder (American Psychiatric Association, 2022). It is true that his self-reports show that some of Jung’s experiences (notably the daytime hallucinations) fall in the category of psychotic symptoms. That said, anomalous experiences such as hypnagogia and psychotic symptoms are quite common in the general population and experiencing psychotic symptoms does not necessarily equate to having a psychotic disorder (Linszen et al., 2022; Ghibellini and Meier, 2023).

Careful analysis of the newly published *Black Books* indicates that a substantial part of Jung’s curious perceptual experiences rather stemmed from hyperphantasia, a condition his contemporaries and later biographers could not take into account because it had not yet been conceptualised and researched. In contrast to hallucinations, it is considered unlikely that a person loses touch with reality while being engaged in their (less clear and distinct) mental imagery. Extremely vivid mental imagery however, is an exception that may well have led prior authors to erroneously interpret Jung’s experiences as being indicative of ‘psychosis’.

Our analysis of Jung’s experiences thus hints at a potential relation between extremely vivid mental imagery and perceptual experiences (notably hallucinations), a relation that Jung himself also suggested a century ago. Taking into account the results of the above-mentioned experimental study by Dijkstra and Fleming (2023), it may well be that those individuals who go on to experience hallucinations either have an altered ‘reality threshold’ or experience an exceptionally high degree of imagery vividness. This underscores the relevance of hyperphantasia for studies in the schizophrenia spectrum, but also in the group of so-called ‘healthy hallucinators’ who run the risk of mistakenly being diagnosed with a schizophrenia spectrum disorder. Along with the Perky effect, in which actual sense perceptions are mistaken for mental imagery (Perky, 1910), the findings of Dijkstra and Fleming (2023) also underline the relevance of the concept of the ‘reality threshold’ and its alleged role in distinguishing between endogenously and exogenously mediated percepts. There is no evidence that Jung and Perky knew each other’s work, but in 1909 Jung appears to have had something similar in mind when he suggested that individuals diagnosed with schizophrenia create a mental ‘reality equivalent’ in which the external world ‘is compensated by a progressive increase in the creation of fantasies, which goes so far that the dream world becomes more real for the patient than external reality’ (Jung, 1960, p. 120). The above-mentioned finding that vivid mental imagery crossing the reality threshold becomes indistinguishable from regular sense perception can perhaps be seen as a computational and neuroscientific corroboration of this idea. Future studies are needed to further investigate this purported mechanism, and to establish its relevance for different groups of people who experience hallucinations, notably those with a psychotic disorder.

Circling back to Jung, it is noteworthy that he himself asserted that psychosis is characterised by ‘a failure to contain and comprehend’ the content of one’s experiences in the context of one’s own life. Jung’s life and work demonstrate that he did manage to interpret the content of his perceptual experiences, to put them into perspective, to find meaning in them, and to share them with others - to great acknowledgement and acclaim.

## Data availability statement

The original contributions presented in the study are included in the article/Supplementary material, further inquiries can be directed to the corresponding author.

## Author contributions

FI: Conceptualization, Investigation, Writing – original draft, Writing – review & editing. JB: Conceptualization, Investigation, Writing – original draft, Writing – review & editing.
