# Supplementary material for: Carl Jung: a life on the edge of reality with hypnagogia, hyperphantasia, and hallucinations
Source: Front Psychol. 2024 Mar 7;15:1358329. doi: 10.3389/fpsyg.2024.1358329 (PMC10954828; doi:10.3389/fpsyg.2024.1358329)
Supplement: Supplementary file 1 [file Table_1.DOCX]

**Supplementary material**

**Carl Jung’s medical history and self-reported anomalous perceptual experiences**

*Abbreviations:*

*BBV: Black Books, volume; MDR: Memories, Dreams, Reflections; RB: Red Book; CW: Collected Works*

**1875 July 26** Born in Kesswil, Switzerland

**Around 1878** Auditory hallucinations: **‘**I would hear things walking about

in the house.’ (MDR22)

General eczema (MDR21)

Fall from stairs and fall against a stove leg, head wound sutured (MDR22)

**1882**  Pseudocroup (MDR32)

**1886** Experience of two autonomous personalities, ‘No.1 and No.2’. Feels

as if he were Dr. Stuckelberger, ‘living in two ages simultaneously, and

being two different persons.’ No.1 is ‘the schoolboy of 1890’ and No.2

‘the other, the old man’ (MDR52, MDR63, MDR113)

**1886** Compulsive thoughts: God leaving a ‘turd’ on a cathedral from His

throne (MDR52-57)

**1887** Fainting spells, possibly epilepsy according to a physician

(MDR47)

**1899 September 12** Visual hypnopompic hallucinations: ‘I suddenly woke up from a dream.

‘It seemed to me as if I was in a dark house, where all kinds of uncanny, ghostly phantoms looked at me. I woke up and discovered that I was awake; I opened up my eyes-completely awake- and saw a faintly illuminated whitish figure beside my bed. The phantom looked like the figure of a very beautiful girl about 10 years old of small stature. The figure hovered, so it seemed, about one meter above the floor. The child had dark loose hair, the face was directed above and sideways, that I could not perceive her physiognomy. A wide, creased, gauze-like garment engulfed her body in a hanging way. The figure hardly stayed a few seconds. Then it seemed to me as if I saw an old woman of big shape, then again, by a statue of marble of an older aged man. My nerves were not at all overexcited this evening. I cannot think of any physiological or psychological reason (BBV2-152/Jung Family Archives pp. 28-29)

**1913** Experienced ‘a state of disorientation’ (MDR205), and ‘suspected there

was some psychic disturbance’ in himself (MDR208)

**1913 October** Hyperphantasia and auditory hallucinations, making him afraid

of becoming psychotic; sees dead bodies and blood floods, hears a voice saying, ‘Look at it well: it is wholly real and it will be so. You cannot doubt it. I asked myself whether these visions pointed to a revolution, but could not really imagine anything of the sort’. Decided that he ‘was menaced by a psychosis’ (MDR209, RB123-124)

**1913 December 12** Hyperphantasia. ‘It was during the advent of the year 1913 - December

12, to be exact - that I resolved upon the decisive step. I was sitting at my desk once more, thinking over my fears. Then I let myself drop. Suddenly it was as though the ground literally gave way beneath my feet, and I plunged down into dark depths. I could not fend off a feeling of panic. But then, abruptly, at not too great a depth, I landed on my feet in a soft, sticky mass.’ (MDR214) ‘‘I am falling,’ a voice said in me,’ in a ‘madness of my own semi-darkness.’ (BBV2-168)

**1913 December 18**  Hypnopompic auditory hallucinations, becomes suicidal: ‘When I

awoke from the dream, I turned it over in my mind, but was unable to understand it. I tried therefore to fall asleep again but a voice within me said, ‘You must understand the dream, and must do so at once’. The inner urgency mounted until the terrible moment came when the voice said: 'if you do not understand the dream, you must shoot yourself!’ In the drawer of my night table lay a loaded revolver, and I became frightened.’ (MDR215-216) ‘After this dream I went through a mental torment unto death. And I felt that I must kill myself if I could not solve the riddle. I knew that I must shoot myself, if I could not understand the dream.’ (BBV2-175). ‘I thought to myself, ‘if this means anything, it means that I am hopelessly off. I had the feeling that I had an over-compensated psychosis, and from this feeling I was not released till August 1st, 1914.’ (BBV1-19)

**1913 December 25** Says that he experiences visual hallucinations *during a dream*: ‘I have

the feeling of a hallucination.’ (BBV2-193)

**1913 December 28** Auditory and visual hypnagogic hallucinations: **‘**Actually it’s uncanny

here - Heaven knows what goes on here - weren’t those steps just now? No, that must have been outside in the corridor - I roll over, firmly closing my eyes - I simply must sleep, wasn't that the door just now? My god. Someone is standing there? Am I seeing straight? A slim girl, pale as death standing at the door. I cannot speak out of fear and wonder. She is coming nearer. ‘Forgive me, but are you real? That you are not simply some unfortunate product of my sleepless brain. My dear child, I believe you, despite all and everything, that you are real. What can I do for you? (...) Forgive me - but are you real? It’s the sorriest likeness to those foolishly threadbare scenes in novels for me to assume that you are not simply some unfortunate product of my sleepless brain.’ (BBV2-206) ‘With these words her shape dissolves into darkness.’ (BBV2-209) Reflects on this experience as: ‘In a lonely house in a wood, where an old scholar is living. Suddenly his daughter appears, a kind of ghost, complaining that people always only consider her as fantasy.’ (CW 9, pt. 1, par. 361)

**1914 January 9** Hyperphantasia: ‘I am basically convinced that Izdubar is hardly real in

the ordinary sense, but is a fantasy. Izdubar will of course not accept that he is a fantasy, but instead claim that he is completely real and that he can only be helped in a real way. ‘I think that you are not at all real, but only a phantasy’. I do not mean to say that you are not real at all, of course, ‘but only as real as fantasy.’ While admittedly he is a fantasy now, the situation remains extremely complex. Even a fantasy cannot be simply negated. It calls for action. Anyway, he is a fantasy, thus considerably more volatile.’ Manages to carry Izdubar’s weight, only after realizing that he is a phantasy. (BBV3-127)

**1914 January 12** Hyperphantasia (or internal verbal auditory hallucinations) in which

he receives commands: ‘The inner voice speaks’ (...) ‘you are cowardly, take out the liver. Why should I do this? This is absurd. I want you to remove the liver. You must do it (her voice becomes threatening). Who are you to give me such an order? ‘Take a piece of the liver, in place of the whole, and eat it.’ What are you demanding? This is absolute madness.’ (BBV3-134)

**1914 May 25** Auditory hypnagogic hallucinations: ‘Then one night**,** I heard the voice

of an old man. He spoke slowly, heavily and his sentences appeared to be disconnected and terribly absurd, so that the fear of madness seized me again. These sentences that the old man spoke to me on the night of the 25 May of the year 1914 appeared to me dreadfully meaningless. ‘This sadness did not leave until the 24th June 1914. From here on the voices of the depths remained silent for a whole year. Again in summer, when I was out on the water alone, I saw an osprey plunge down not far from me; he seized a large fish and rose up into the skies again clutching it. I heard the voice of my soul and she spoke: That is a sign that what is below is borne upward. Soon after this on an autumn night I heard the voice of an old man (and this time I knew that it was Philemon).’ (RB 472-3)

**1914 June 24**  Hyperphantasia: ‘I hear peculiar words inside me, such as ‘You are

silly, stupid, deluded’, ‘You are your own ape, a ball in the hands of

others.’ (BBV5-234)

**1914 August**  Increase in frequency, intensity, and complexity of a range of

experiences (e.g. hyperphantasia, hallucinations, personifications, and dreams): ‘And an incessant stream of fantasies had been released, and I did my best not to lose my head but to find some way to understand these strange things. As soon as I had the feeling that I was myself again, I abandoned this restraint upon the emotions and allowed the images and inner voices to speak afresh’. (...) ‘Sometimes it was as if I were hearing it with my ears, sometimes feeling it with my mouth, as if my tongue were formulating words, now and then I heard myself whispering aloud. Below the threshold of consciousness, everything was seething with life.’ (...) ‘I felt not only violent resistance to this, but a distinct fear. For I was afraid of losing command of myself and becoming a prey to the fantasies and as a psychiatrist I realized only too well what that meant.’ (MDR212-214)

Personification: ‘The essential thing is to differentiate oneself from the unconscious contents by personifying them, and at the same time to bring them into relationship with the consciousness. That is the technique for stripping them of their power. It is not too difficult for them personally, as they always possess a certain degree of autonomy, a separate identity of their own.’ (MDR223) ‘Philemon represented a force which was not myself. In my fantasies I held conversations with him, and he said things I had not consciously thought. For I observed clearly that it was he who spoke.’ (MDR219) ‘When I was writing down these fantasies, I once asked myself, ‘What am I really doing?’ Certainly this has nothing to do with science/ but then what is it? Whereupon a voice within me said, ‘It is art.’ I was astonished. It had never entered my head that what I was writing had any connection with art. I knew for a certainty that the voice had come from a woman. I recognized it as the voice of a patient, a talented psychopath who had a strong transference to me. She had become a living figure within my mind. (..) I said very emphatically to this voice that my fantasies had nothing to do with art, and I felt a great inner resistance. No voice came through, however, and I kept on writing. Then came the next assault, and again the same assertion: ‘That is art.’ This time I caught her and said, ‘No it is not art! On the contrary, it is nature.’ and prepared myself for an argument. When nothing of the sort occurred, I reflected that the ‘woman within me’ did not have the speech centres I had. And so I suggested that she use mine. She did so and came through with a long statement. I was greatly intrigued by the fact that a woman should interfere with me from within.’ (MDR221)

**1916** Auditory and visual hallucinations, sensed presence: ‘It began with a

restlessness, but I did not know what it meant or what they wanted of me. There was an ominous atmosphere all around me. I had the strange feeling that the air was filled with ghostly entities. Then it was as if my house began to be haunted. My eldest daughter saw a white figure passing through the room. My second daughter, independently of her elder sister, related that twice in the night her blanket had been snatched away and that same night my nine-year-old son had an anxiety dream.’ (...) ‘Around five o'clock in the afternoon on Sunday the front door bell began ringing frantically. It was a bright summer day, the two maids were in the kitchen, from which the open square outside the front door could be seen. Everyone immediately looked to see who was there, but there was no one in sight. I was sitting near the doorbell and not only heard it but saw it moving. We all simply stared at one another. The atmosphere was thick, believe me! Then I knew that something had to happen. The whole house was filled as if there were a crowd present, crammed full of spirits. They were packed deep right up to the door, and the air was so thick it was scarcely possible to breathe. As for myself, I was all aquiver with the questions for God’s sake, what in the world is this? Then they cried out in chorus. ‘We have come back from Jerusalem where we found not what we sought. That is the beginning of the septem sermones.’ I saw that so much fantasy needed firm ground underfoot, and that I must first return wholly to reality. For me reality means scientific comprehension. It is, of course, ironical that I, a psychiatrist, should at almost every step of my experiment have run into the same psychic material which is the stuff of psychosis and is found in the insane. This is the fund of unconscious images which fatally confuse the mental patient. But it is also the matrix of a mythopoeic imagination which has vanished from our rational age. Though such imagination is present everywhere, it is both tabooed and dreaded, so that it even appears to be a risky experiment or a questionable adventure to entrust oneself to the uncertain path that leads into the depths of unconsciousness. It is considered the path of error, of equivocation and misunderstanding.’ (MDR224-226)

**1916 June 1**  Visual and auditory hallucinations: ‘It was noon on a hot Summer’s day

and I was taking a stroll in my garden; when I reached the shade of the high trees, I met Philemon strolling in the fragrant grass. But when I sought to approach him, a blue shade (identified as Christ, see BBV6-85) came from the other side…’ ‘The shade answered…’ (RB551-552)

**1917 October 7** Auditory and visual (and possibly tactile) hallucinations, with

personification: ‘I was writing in my book and suddenly saw a man standing watching over my shoulder. One of the gold dots from my book flew up and hit him in the eye. He asked me if I would take it out. I said no - not unless he told me who he was. He said he would. You see, I knew that. If I had done what he asked then he would have sunk into the unconscious and I would have missed the point of it, i.e.: why he had appeared from the unconscious at all. Finally, he told me that he would tell me the meaning of certain hieroglyphics which I had had a few days earlier. This he did and I took the thing out of his eye and he vanished.’ (BBV7-149)

**1917 March 29**  Colitis mucosa (BBV6-283)

**1918 February 15** Bronchitis (BBV7-174)

**1918 February 20** Pertussis (weeping cough) for a week (BBV7-175)

**1918 September 14**  Recurrence of pertussis (BBV7-182)

**1919 April 30**  Hypnagogic hallucinations (according to Jung himself): ‘There beside

me on the pillow, I saw the head of an old woman, and the right eye, wide open, glared at me. The left half of the face was missing below the eye’. In England staying in a haunted house: First saw the left half of a face of an old woman. Then all sorts of sounds. Paralysing fear, sleeplessness, suddenly subsiding in the other room. Then saw Toni half materialized. ‘The vision had the character of a hypnagogic hallucination and was probably a reconstruction of the memory of the old woman with carcinoma.’ (BBV7-199)

**1919 July 21** Spanish flu, 40 degrees fever, delirium with visual hallucinations of

geometrical figures, spheres (BBV7-200)

**1919 August** Hyperphantasia: ‘Vision in the same night. To the right of the bed of

my wife there is a great angel of the most severe form in a praying

position. To the left of him is a dark transparent dangerous mass. I see only the following forms in it. Then I see the angel to the left of me. He indicates a bright spot from which a deathly pale maiden with almost closed eyes, black hair, sharp southern features, around 28 years old steps forward. She remains around 2 meters from my bed and excites an uncomfortable sexual feeling in me.’ (BBV7-201)

**1922 January 23-27** Flu (BBV7-219)

**1923 January 12** Either visual, auditory, and musical hypnagogic hallucinations, or

hyperphantasia: ‘This evening I slept a bit at 6 o'clock. Before sleeping I suddenly saw the face of my father, very vividly. He smiled and seemed to be in a very good mood. At 11 o'clock at night on the same day before sleeping I saw my mother, somewhat as I saw her in the coffin, floating upward out of the darkness, her head slightly bent back, her eyes closed, as if sleeping or in a swoon, but living inside. My father stood right next to her, smiling, in another state, he looked at her smilingly. Musical hallucinations: ‘During the entire journey I continually heard dance music, laughter and jollity, as though a wedding were being celebrated.’ (BBV7-232)

**1923 January 13** Visual (possibly grief) hallucinations: ‘I did have very ominous

premonitory experiences and dreams. For example, my father, who has been dead for 27 years, appeared in a dream, and also once when I was awake, and he was laughing and in a good mood.’ (BBV7-233)

**1923 February 8** Auditory (hypnopompic) hallucinations and sensed presence: ‘They

came in the night. Many in an invisible horde. They drifted past my house. I woke up when they went past. I went out to see and didn't know where they came from and where they were going. They came from the east and wandered to the west. On both sides they streamed past my house. I heard them stumble over the big stones.’ (BBV7-234)

**1927 January 13** Hyperphantasia: could barely distinguish between reality and fantasy.

‘A vivid feeling the night after, as if he waved to me, standing at the foot of my bed. I decided to follow him (in thought).’ ‘One night I lay awake thinking of the sudden death of a friend whose funeral had taken place the day before. I was deeply concerned. Suddenly I felt that he was in the room. It seemed to me that he stood at the foot of my bed and was asking me to go with him. I did not have the feeling of an apparition rather, it was an inner visual image of him, which I explained to myself as a fantasy. But in all honesty, I had to ask myself, ‘Do I have any proof that this is a fantasy? Suppose it is not a fantasy, suppose my friend is really here and I decided he was only a fantasy - would that not be abominable of me?’ Yet I had equally little proof that he stood before me as an apparition. Then I said to myself, ‘Proof is neither here nor there! Instead of explaining him away as a fantasy, I might just as well give him the benefit of the doubt and for experiments’ sake credit him with reality.’ The moment I had that thought, he went to the door and beckoned me to follow him. So I was going to have to play along with him! That was something I hadn't bargained for. I had to repeat my argument to myself once more. Only then did I follow him in my imagination.’ (BBV7-240)

**1927 May 23**  Dyshidrosis of the hands​​ (BBV7-242)

**1944**  Foot fracture and myocardial infarction, followed by delirium with

limited reality testing and depersonalization: ‘It seemed to me that I was high up in space’, ‘while I floated in space’. Gets angry at his physician because he was ‘stubbornly refusing to speak of all that had passed between us in my vision’. He has three ‘visions’ that ‘lasted for about an hour’. **‘**It was not a product of the imagination. The visions and experiences were utterly real.’ ‘There was nothing subjective about them; they all had a quality of absolute objectivity. They were nontemporal.’ ‘In which present, past and future are one.’ (MDR341-347)

**1946 November 4** Second myocardial infarction, refuses hospitalization and remains ill

for three months

**1961 May 17** Stroke: brain embolism with transient aphasia

**1961 May 30** Stroke: second brain embolism

**1961 June 6** Dies in Küsnacht, Switzerland
